# Sink or Swim: Virtual Life Challenges among African American Families during COVID-19 Lockdown

**DOI:** 10.3390/ijerph18084290

**Published:** 2021-04-18

**Authors:** Adaobi Anakwe, Wilson Majee, Kemba Noel-London, Iris Zachary, Rhonda BeLue

**Affiliations:** 1 College for Public Health and Social Justice, Saint Louis University, St. Louis, MO 63104, USA; 2 Department of Health Sciences and Public Health, University of Missouri, Columbia, MO 65211, USA; majeew@missouri.edu; 3 Department of Occupational Therapy, University of the Western Cape, Western Cape, Bellville 7535, South Africa; 4 Department of Health Management and Policy, Saint Louis University, St. Louis, MO 63104, USA; kc.noellondon@slu.edu (K.N.-L); rhonda.belue@slu.edu (R.B.); 5 Health Informatics, University of Missouri, Columbia, MO 65211, USA; ZacharyI@health.missouri.edu

**Keywords:** COVID-19, African American, emotional health, family engagement, digital technology divide

## Abstract

This study explores African American parents’ experiences with using technology to engage their children in meaningful activities (e.g., e-learning) during COVID-19 and its impact on family health. Eleven African American families were recruited through a local health department program from a rural Midwestern community to participate in semi-structured interviews. Majority of participants reported stresses from feelings of “sink or swim” in a digital world, without supports from schools to effectively provide for their children’s technology needs. The COVID-19 pandemic underscored the importance of family-school collaborative engagement and empowerment. Digital technology needs to become part of our school education system so that technology use among African Americans is elevated and families protected against future outbreaks. Further research with a more diverse African American sample is needed.

## 1. Introduction

The unprecedented emergence and global spread of the SARS COV-2 virus (COVID-19) has resulted in severe social and economic consequences for families globally. Millions across the world have lost their loved ones to COVID-19 [1,2], suicide rates have increased as unemployment and financial instability sets in as a result of the pandemic [3,4,5]. Interpersonal violence risks have been elevated, particularly among those subjected to quarantine [6,7]. In the U.S., the COVID-19 pandemic has accentuated the racial health disparities that have plagued the U.S health system for decades. For example, in Wisconsin, African Americans make up 6% of the population yet endured 50% of the COVID-19 deaths. Similarly, only 30% of Chicago’s population is African American, yet 70% of the COVID-19 deaths were reported in this population [8]. African Americans continue to be disproportionately affected by underlying root causes such as structural racism and economic inequalities which contribute to gaps in health insurance coverage, uneven access to services (schools, housing, transportation, healthy food, green spaces, recreational facilities, etc.) [9,10,11]. Over time, these social determinants of health have resulted in higher rates of chronic medical conditions, including obesity, diabetes, and kidney disease—all of which are risk factors for severe illness from COVID-19 among African Americans. Not only are African American families at increased risks for contracting and dying from the virus, but they are also more likely to endure more economic strain and hardship due to job losses, pay cuts and single parent household status.

In response, governments as well as local, national, and international organizations, have taken measures to decelerate the transmission of the virus, and reduce the number of deaths associated with it [12]. One such measure was to close physical matriculation in schools and shift to a virtual/online model. According to the United Nations Educational, Scientific and Cultural Organization [13], an overwhelming majority (90%) of the world’s enrolled students in nearly 200 countries experienced temporary school closures. This change forced some students to adapt to online learning or for others, particularly those in rural areas, to completely miss school [14]. Although school closures were found effective in transmission reduction during previous influenza outbreaks, including the Swine Flu [15], they can have unintended long-term negative impact on children and families [14]. Studies have begun to demonstrate that COVID-19 related school closures have unprecedentedly altered lives of the students, their families, and their educators in significant ways (e.g., increased stress among family members due to prolonged times together among limited resources—space, Wi-Fi, play stations, etc.) (Authors Blinded) [16].

Further, the COVID-19 pandemic has highlighted one key unresolved issue related to technology tools, the “digital divide”, which refers to the growing gap in internet access between the underprivileged members of society, especially the urban and rural poor, the elderly, and children and adults with disabilities who do not have access to computers, the internet, or digital resources and the urban and suburban wealthy population who have access [17]. An Educause COVID-19 QuickPoll conducted in Australia, Canada, Colombia, Finland, India, South Africa, Thailand, United Kingdom, and the U.S. reported that students from 36% of the 267 institutions polled experienced moderate or extreme difficulty getting the bandwidth and/or Wi-Fi, which is necessary for continuing academic work [18]. Of the 267 respondents, 248 (93%) represented U.S. institutions. Although the Black/White digital divide in the U.S. is not new, the pandemic has threatened to widen this gap as stark disparities in access to broadband/Wi-Fi persists. Many African Americans have reduced access to technologies that allow their children and families to have equitable access to education while at home [19]. In a survey of technology access among all U.S. households during the pandemic, Ong (2020) found that African American (36%) and Hispanic (37%) households lagged far behind their White (28%) and Asian (24%) counterparts, which mirrored the income disparities between these populations (Ong, 2020) [20].

In another study, responses from teachers on a question about the biggest instructional challenges during pandemic-related school closures suggest that their ability to communicate with students and their families was often constrained by students’ lack of internet or appropriate technologies at home (e.g., devices). Only 30% of teachers in high-poverty schools (which are disproportionately minority schools) reported that all or nearly all their students had access to the internet at home, compared to 83 percent of teachers in low-poverty schools [21]. According to Pew Research (2020) roughly one-in-five parents with homebound schoolchildren reported that it is very or somewhat likely their children will struggle with schoolwork because of lack of access to a computer at home (21%) or have to use public Wi-Fi to finish their schoolwork because there is not a reliable internet connection at home (22%) [22]. These gaps can exacerbate the disparities that exist in educational outcomes, particularly for low-income African American families. For example, it was demonstrated on Advanced Placement tests, where a score of 3 or above is considered “successful”, that while 62–64% of White and Asian students’ scores were considered successful, approximately 43% of Latino and American Indian students are successful, and only 26% of Black students who took the test had scores considered successful [23].

Lack of appropriate learning technologies and devices impacts how parents engage with their children and teachers on online learning. Parental involvement and support are critical for successful student learning [24,25]. However, with mandatory COVID-19 school closures, parents were thrust into unfamiliar roles and responsibilities such as being facilitators, pseudo-teachers, and coaches, as their children participate in virtual learning [26]. Many teachers, families, and learners were unprepared for this sudden shift. Parents had to assist their child(ren) at various academic levels with different types of distance learning (from providing the resources in the home such as Wi-Fi and computers, to navigating learning through these “new” online platforms). As a result, parents struggle with understanding, redefining, and relearning what roles they should play in their children’s online learning [27]. Challenges, including limited economic resources, inadequate internet access, and reduced digital self-efficacy [28,29] may individually or collectively impact the level of parental involvement in e-learning settings.

Parental burnout, a stress-related disorder that is defined as “a prolonged response to chronic and overwhelming parental stress” [30] (p. 1319), can have far reaching impacts on family relations and child outcomes. This condition occurs in about 5 to 20% of all parents [31]. During the pandemic, parents were at increased risk for experiencing burnout due to increased demands on their time and involvement with their children while also managing their own stresses and anxieties [32]. These fears and anxieties can be overwhelming and cause strong emotions in both adults and children. Further, as a total dependence on technology became the primary form of engagement, the persistent feelings of isolation and loneliness, especially among families that lacked access to reliable internet, have been connected to poor health outcomes such as stress and anxiety [33,34,35].

Although the world is awaiting the vaccination process to take root, many African American families continue to grapple with the new normal of a virtual learning environment for their children. This study explored African American parents’ experiences with using technology to engage their children during COVID-19 and the impact on family health. Further, as more research emerges on parental burnout during the pandemic, this study sought to provide a nuanced understanding of how access to and usage of technology can contribute to parental stress. In this study, we use the terms virtual learning, online learning, and e-learning synonymously.

## 2. Methods

### 2.1. Setting and Participant Selection

The study protocol was approved by the institutional review board at the lead author’s university. The study used an exploratory, descriptive, contextual qualitative design to collect information on the technological experiences of African American families during COVID-19. According to Creswell (2013), to study a problem, qualitative researchers use an emerging qualitative approach to inquiry and the collection of data in a natural setting sensitive to the people and places under study [36]. Burns and Grove (2003) define exploratory research as research conducted to gain new insights, discover new ideas, and/or increase knowledge of a phenomenon. A contextual design refers to and focuses on specific events in “naturalistic settings” (p. 32) [37]. The study recruited 13 African American families (Table 1), with at least one school-aged child (5–17 years), through the community’s local health department (LHD) from the rural Midwest. Purposive and snowball sampling methods were used to reach populations that are otherwise difficult to reach [38]. With the help of the LHD, participants who expressed interest in the study provided their contact information to the lead author and were briefed on the purpose of the study. Those interested scheduled a time for a telephone interview. In total, 13 families expressed interested of which 11 interviewed. The inclusion criteria were (a) parent or guardian living with at least one school-aged child age between 5–17 years and (b) availability to interview by phone or Zoom. Verbal consent was obtained from all participants. No incentives were provided for participation.

### 2.2. Data Collection

Semi-structured phone interviews were used to collect data. Interview questions were formulated in three phases. First, two researchers (AA and WM) drafted the questions based on the literature and study objectives. Second, the questions were reviewed by one researcher (RB). Third, AA and WM compiled the final list of questions. Interviews were conducted by one researcher (AA) and, on average, lasted about 45 min (range 30 to 60 min). The semi-structured interview guide (Table 2) consisted of seven questions that broadly examined African American families’ awareness of COVID-19 and the strategies they were using to cope with the pandemic. Data collection continued until saturation was reached (i.e., the point at which no new useful information was generated) [39,40].

### 2.3. Data Analysis

All interviews were conducted by the lead author and audio recorded. Recordings were transcribed verbatim by a professional transcriber. Data were thematically analyzed by two researchers (AA and WM) [41]. According to Braun and Clark (2006), thematic analysis is used for “identifying, analyzing, and reporting patterns within the data” (p. 79). Data analysis followed four phases: familiarization with data, code development, code refinement, and revision of codes into themes [41]. First, AA and WM familiarized themselves with the data and independently coded four transcripts. Second, the two researchers discussed the thoughts, codes, and themes that emerged from the four transcripts. Through this inductive process, the researchers developed a coding scheme [41] that was used for the other seven transcripts. One researcher (AA) completed the rest of the coding. Third, AA and WM reviewed the coded transcripts together to verify complete and reliable coding. Interrater reliability of 84% was achieved. Fourth, the two researchers mutually resolved any areas of disagreement. Further analysis was done in Dedoose software, Version 8.0. 35Los Angeles, CA, USA [42].

The three authors (AA, WM, and RB) who were involved with research design, data collection, and analysis were all of African descent. AA is an African-born female graduate student, WM is an African-born male researcher with expertise in the field of qualitative research, and RB is an African American female scholar. The three researchers were critically self-reflective about their own lived experiences, preconceptions, relationship dynamics, and analytic focus on the processes by which interview questions were formulated and data were collected, analyzed, and presented [43,44]. As Black researchers working with an African American community, their shared identity with the community was central to building trust and rapport. Further, the diverse backgrounds (age, gender, sociocultural experiences) and research training of the team added richer perspectives to interpreting and contextualizing study findings. Having one researcher conduct all interviews and immediately discuss them with the other researchers helped in revising questions and developing probes, thereby reducing biases that are associated with having one data collector in exploratory studies. Finally, having an African American review both data collection and manuscript preparation, provided insights from a lived experience perspective that strengthened the study.

## 3. Results

### 3.1. Emerging Themes

Three major themes emerged around familial adjustment to the new realities of COVID-19: (1) sudden and drastic changes to online platforms, (2) familial engagement challenges, and (3) technology use for learning and leisure. The “familial engagement challenges” theme had two sub-themes: sharing of resources and lack of Wi-Fi/internet.

### 3.2. Sudden and Drastic Changes—Sink or Swim

Many families appreciated use of internet in helping them to adjust to the challenges created by COVID-19. One participant expressed, “*Thank God for the internet because other than that I wouldn’t know what to do.” (032)*. Another participant acknowledged the important role the internet plays in life, yet felt that going virtual had been too sudden and drastic. They stated,

Well, social media is a blessing and a curse. We understand that we can have grocery stores and we can have restaurants, we can have all this but guess what, we don’t have to leave our house if we don’t want to, for nothing. It can all be delivered and we can still be connected socially, virtually and so before this we was kind of tiptoeing into it. Now we’re going virtual…. now everybody’s been dumped into it and say, sink or swim. (038)

Sudden and drastic changes can be stressful and even anxiety-producing as families struggled to find ways to adjust to living arrangements that were changing drastically. Students no longer had access to the computers and Wi-Fi at school. Families who were accustomed to their children spending the day at school had to deal with finding activities to engage them in ways that fostered resource sharing. Although parents observed that their children were more engaged with activities delivered through their electronic devices, e.g., phones, tablets, and computers, they also were mindful of the need for less screen time. However, because the switch to mostly internet/online based engagement was sudden, families felt they were not prepared to engage their children otherwise. One participated noted,

*I fully supported the decision to cease that* (excessive use of devices) *…so where we used to have like screen time and turn the TV up and don’t look at your electronic all the time. But I started to be relaxed in all that moreso than ever because, goodness, they don’t have anything else, no other way to connect with their friends. (037)*

### 3.3. Familial Engagement Challenges

Although availability of internet provided relief for many families in terms of adapting activities to engage children, it created two main challenges for low-income families, (a) fighting over limited electronic gadgets/devices such as access to phones, tablets, TV, and computers and (b) limited Wi-Fi connectivity.

#### 3.3.1. Fighting over Resources

Because of spending a lot of time together with little non-social media or digital leisure activities, children started to pick at each other over little things, “*They start snapping at each other, arguing over things they normally wouldn’t argue over, like the Play Station. They would argue over whose turn it was to play on that.*” (040). And in many cases, “*Just the 10-year-old wanting to be in his older brother’s room or wanting to play his video games and the older brother not wanting to let him play because he’s usually gaming online with other people.*” (039) Summing up their feelings over how the sudden and drastic changes due to COVID-19 pandemic had affected their family, participants noted,

My children are fighting more, not like they physically fight… “Well, I’m watching this” (on the tablet). That’s a fight every day. Like, I have to minimize the times that the kids use it. Then you don’t want them to be on devices too long so I’m up to the point where taking them and hiding them up under my mattress. You got an 8-year old and you got a 10-year old and you have to work with them and then they fight amongst each other, then they fight with the 16-year old. They drive me insane. (032)

It happens a lot when I’m doing something like cooking or cleaning or something my daughter will just randomly start picking with my son for whatever reason, like “Give me the phone” or “Let me watch YouTube” or something like that and then my son gets easily annoyed, then he be like “You’re so annoying. Stop,” or something like that, and then that’s when it’s time to be like “Okay, guys, stop, separate yourselves.” (031)

#### 3.3.2. Lack of Access to Wi-Fi

For some families, inability to have internet at home was a big challenge and parents felt that in unsettling times like during the COVID-19 pandemic when life revolved around internet, such a resource should be provided by government to all for who are not able to afford it. One participant said,

I think that (internet) should be a given during the school year, period, regardless of what’s going on because we send these kids home with tablets or whatever, laptops, but if they can’t get on the internet to access the things what good is having the tablet at home? (039)

### 3.4. Technology for Learning and Leisure Engagement

Faced with school closures due to COVID-19 global pandemic, the videoconferencing software platform Zoom quickly became the standout resource for schools as they switched to remote classes. Participants acknowledged that having Zoom has helped them and their children stay connected with teachers and church leaders. One participant said, “*Well, my daughter, she gets on Zoom calls with the church so that kind of keeps them connected. The church sent them out face masks and they did like a Zoom face mask session*.” (040) For school work, “*…. the girls had their electronics so they were able to participate in the Zoom calls that the teachers would do once or twice a week*” (037) and “… *like my son’s teacher, they did Marco Polo videos where you can record a video to a group and they can see it later and they can record a video and send it back.*” (039) Detailing her experience, one of the participants expressed,

When school was still in session (from home due to COVID-19) he had one to three sessions four days a week. He was constantly pretty much on Zoom most of the day… If he had needed help outside of his Zoom sessions then, I would help him with that but thankfully they pretty much worked through whatever the assignments were during their sessions so there wasn’t a lot of “teaching” that I needed to do outside of those sessions. (039)

Beyond learning, whether through the schools or church, families engaged their children in technology-based activities such as face time socialization with friends on Zoom and/or Tik-Tok, watching YouTube videos, and playing app-based games on phones or tablets. One participant noted, “*I feel like my kids were really engaged when the things were coming through their devices, their activities, the things that they could do, the fun stuff, the engagement.*” (037) Another parent acknowledged,

But the kids, they’ve been playing Roblox. That’s the number one app they’ve been playing. Most of the kids have also been watching YouTube videos. Cocomelon YouTube Video did a billion streams in one week last week. (038)

It was interesting to further observe that parents engaged their children in decision-making processes around how to keep them engaged. Much of the desire to involve children in decision-making stemmed from the fact that parents did not want their children overwhelmed by the sudden and drastic changes. Involving them would ease the frustration of “sinking or swimming” with regards to the sudden changes caused by COVID-19, particularly sheltering in places and going virtual, as pointed out by one participant,

I feel like we did good for what we had, like having the kids be a part of the planning process of what our days would look like, keeping them involved, like having our little family meetings, so keeping them involved, the best way we could without scaring or overwhelming them. (037)

Participants also noted the role teachers played in keeping their children engaged while sheltering in place. A parent noted, “They had their homework assignments…the teacher would send her messages, to check on her to see if assignment wasn’t done and stuff like that.” (041). A grandparent also said,

My granddaughter will get on the internet one to three days out of a week. She did it with her school and with Grade A Plus. They both got on the internet and the teacher would do activities on the internet on Zoom. (036)

## 4. Discussion

This study explored African American families’ experiences with using technology to engage their children during COVID-19 and how that impacted family health. Understanding these experiences and perceptions can aid the development of solutions that address communication and relational challenges at familial level, and strengthen the collaboration between parents, teachers, and students. Based on our analysis, many parents experienced emotional stressors from navigating sudden changes—social distancing, business and school closures, and stay at home mandates. Similar to studies that observed parents’ mixed feelings about online learning—with some feeling more connected to their child’s schoolwork while others felt more burdened [45]—evidence from this study echoed similar experiences.

African American parents already deal with a disproportionate COVID-19 burden and limited access to reliable Wi-Fi. Coping with the challenges of supporting their children in this new learning environment can be stressful to parents. Based on the interviews, parents felt they were placed in a survival mood in which they had to either “sink or swim” with regards to understanding their own limitations with technology and the needs of their children to effectively support them. Prior to the pandemic, parents were tasked with reducing screen time for their kids and involving them in more physical activity [46]. In a pandemic world, however, parents were not only tasked with “reversing” that messaging but also becoming more involved with technology themselves and for their kids. Findings from our study showed that not only do African American families lack access to technology, but they also grapple with how to keep their children engaged without seemingly providing inconsistent messaging on use of technology. On the other hand, parents also had to bear the additional burden of sharing “limited” technology resources. Nevertheless, parents had to navigate their moral and ethical beliefs and the situational conundrum with minimal support, which can increase anxieties due to feelings of powerlessness, lack of control and disempowerment with navigating this “new” tech world safely. Many parents (a) lacked the technical expertise with the technologies their children were using such as Zoom and in the material children were learning and (b) had no access to training and support from professionals. Some parents lacked dependable broadband/Wi-Fi. These factors contributed to parents’ stress as they were caught in between the desire to support their children and the reality of their limited technological knowhow, and socio-economic status to provide reliable internet. The realization that their poor knowledge of and skills on the virtual platforms their children were using were hindering their participation suddenly became a stressor. Technology can frustrate and stress parents when their children fail to participate in e-learning because of the unreliability of the home internet and technical devices that are available [47,48].

Although technology had its challenges, parents appreciated that when well supported (e.g., with reliable internet, technical training), it can improve both the online learning experience for them, their children and teachers. It can also provide opportunities for family engagement activities such as watching You Tube movies and chatting with other friends or family members on Zoom. Parents felt that where teachers engage with their children in regular and meaningful meetings, it was easier for them to support their children as they (parents) worried less about whether their children were finding their assignments or understanding their lesson plan. Despite the challenges, this can also be a great opportunity to educate parents and educators on how to overcome the digital divide. In other words, COVID-19 driven online learning has provided new opportunities (e.g., flexibility in learning, utilization of different online platforms—WhatsApp, Zoom, Google Teams) to innovatively engage students, particularly generation Z [49,50]. This suggests that collaboration between schools and families is critical to support and meet online learner’s needs effectively and reduce anxiety and stress among parents. Collaboration has the potential to improve the parental engagement experience, especially with effective use of technology.

Families benefit from time together and time apart. However, when resources (e.g., no internet or not enough computers/iPads) are limited, conflict among siblings and between children and parents can occur. This conflict can result in pandemic burnout in which family members become intolerant to each other because of prolonged episodes of sharing space and resources evidenced by the “infighting among children” as they wrestled over access and use of the available electronic gadgets. Even though debates around digital inequities and closing the digital divide are not new [51,52], these inequities were further exacerbated by the rapid changes brought about by the COVID-19 pandemic. This study highlights how digital access or the lack thereof exacerbated parents’ feelings of stress. Living together, continued space sharing and additional responsibilities brought on by the pandemic can increase the risk for parental burnout, an emerging and pertinent mental health issue with cascading adverse effects on family health [32]. However, when all familial experiences of stress are grouped under one “stressors” umbrella, it disallows a nuanced understanding of pathways and contributors to burnout in familial settings and how interventions should be tailored to meet the needs of families in this time. As Locks and colleagues noted, beyond primary stressors (i.e., through experiencing the direct pandemic or disaster effects like death or morbidity), secondary stressors can have a wider reaching negative effect on parental stress and mental health [53].

## 5. Conclusions and Possible Future Research Directions

Although this study is based on a small sample of African American families, it suggests that the U.S. needs to aggressively address the Black/White digital divide, especially to ensure equitable access to academic resources and opportunities for African American communities. In a rapidly digitalizing world, reduced access to these resources can have irreversible long-term consequences for academic, social, and health outcomes. These families need tailored supports to ensure that both their need for and concerns about technology use are addressed, especially as learning through online means gradually becomes the norm. Digital technology needs to become part of the school education system so that technology use and achievement standards among African Americans are elevated. Researchers, community leaders, and local and national governments need to collaborate, especially with technology developers, to create and offer ongoing public educational programs aimed at giving parents and caregivers the technology knowledge and skills they need to remain relevant in a rapidly digitalized world.

Given the insights gleaned from this pilot study, there are four main areas that future research can address. First, there is need for community-based participatory research that broadly investigates how and why minority populations continue to be left behind in the digital world. An important issue to address would be how to prevent the digital divide from becoming another social determinant of health. Second, research should also explore strategies to create and sustain digital safety nets for resource limited families, especially African American communities, to strengthen pandemic recovery. Third, research should address digital inclusion, especially identifying how key educational stakeholders (parents, students, schools, and governments) can work with technology companies to provide context-sensitive culturally relevant technologies to promote family engagement and support students’ success in resource-limited communities. Seamans and Bytes (2020) argued for local and regional policy solutions, including policies that allow communities to provide their own broadband or tax breaks to incentivize employers who provide access to their employees, to close the digital divide [54]. Future research should explore how these policy-level interventions can be implemented. Finally, there is need for research that investigates the impact of increased screen time on children and familial relations, and how to restructure practical and enforceable messaging in a post-pandemic world.

## Figures and Tables

**Table 1:** Demographic characteristics of study participants.

Characteristics	Study Population *n* (%)
Total population	11
Median age, years	43.5
Educational status (25+) ^a^	
High School	2 (16.7)
Some college	7 (58.3)
College or more	3 (25.0)
Role	
Mother	7 (63.6)
Father	1 (9.1)
Grandmother	2 (18.2)
Couple	1 (9.1)
Union status	
Married	1 (9.1)
Separated/Divorced/widowed	4 (36.4)
Not married	1 (9.1)
Never married	5 (45.4)
Change in income	
Increased	1 (9.2)
Reduced	5 (45.4)
Stayed the same	5 (45.4)
Number of children 5 to 17 years old	
None ^b^	1 (9.1)
1	3 (27.3)
2	4 (36.4)
3	1 (9.1)
4	2 (18.1)
Age of parent (categories)	
25 to 35 years	2 (18.2)
36 to 45 years	2 (18.2)
46 years or more	7 (63.6)
Age of children median (interquartile range)	11 (9.0, 16.0)

Notes: ^a^ *n* = 12 to account for the education status of the couple; ^b^ Had two children in the household (24 and 3 years old). Not included in study findings.

**Table 2:** Semi-structured interview guide for community health workers.

Interview Questions
When you think about COVID-19, what comes to mind? How did you hear about COVID-19? What was your reaction to lockdown, sheltering in place, and social distancing? What has been your experience sheltering-in-place? Can you give me an example of the worst experience you have had as a family? What has been your engagement experience with your family? Can you explain some of the activities you have adapted as a family? What has been the challenges/barriers implementing the activities with your school-age children. What has been your experience utilizing shared resources? How has your children participated in this sharing? What has been your experience of connectedness with your family? Explain the ways in which sheltering in place has impacted your family. When you think about coping during this pandemic, what comes to mind? If you were o share one or two examples of successful coping strategies you have used as family, what would those be? If some strategies were not success, why were they not successful. What are your thoughts on engaging your children during this season? What can be done? What could have been done?

## Data Availability

The data presented in this study are available on request from the corresponding author. The data are not publicly available due to maintaining IRB compliance with study protocols.
